# S100A8 and S100A9 Are Associated with Doxorubicin-Induced Cardiotoxicity in the Heart of Diabetic Mice

**DOI:** 10.3389/fphys.2016.00334

**Published:** 2016-08-05

**Authors:** Xiao M. Pei, Bjorn T. Tam, Thomas K. Sin, Feng F. Wang, Benjamin Y. Yung, Lawrence W. Chan, Cesar S. Wong, Michael Ying, Christopher W. Lai, Parco M. Siu

**Affiliations:** ^1^Department of Health Technology and Informatics, Faculty of Health and Social Sciences, Hong Kong Polytechnic UniversityHong Kong, China; ^2^Department of Integrative Biology and Pharmacology, University of Texas Health Science Center at HoustonHouston, TX, USA

**Keywords:** doxorubicin, type 2 diabetes mellitus, cardiotoxicity, S100A8, S100A9

## Abstract

Cardiomyopathy is a clinical problem that occurs in the hearts of type 2 diabetic patients as well as cancer patients undergoing doxorubicin chemotherapy. The number of diabetic cancer patients is increasing but surprisingly the cardiac damaging effects of doxorubicin, a commonly used chemotherapeutic drug, on diabetic hearts have not been well-examined. As the signaling mechanisms of the doxorubicin-induced cardiomyopathy in type 2 diabetic heart are largely unknown, this study examined the molecular signaling pathways that are responsible for the doxorubicin-induced cardiotoxicity in type 2 diabetic hearts. Male 14- to 18-week-old db/db mice were used as the type 2 diabetic model, and age-matched non-diabetic db/+ mice served as controls. The db/+ non-diabetic and db/db diabetic mice were randomly assigned to the following groups: *db/*+CON, *db/*+DOX-5d, *db/*+DOX-7d, *db/db*CON, *db/db*DOX-5d, and *db/db*DOX-7d. Mice assigned to doxorubicin (DOX) group were exposed to an intraperitoneal (i.p.) injection of DOX at a dose of 15 mg/kg to induce cardiomyopathy. Mice in control (CON) groups were i.p. injected with the same volume of saline instead of DOX. Mice were euthanized by overdose of ketamine and xylazine 5 or 7 days after the DOX injection. Microarray analysis was adopted to examine the changes of the whole transcriptional profile in response to doxorubicin exposure in diabetic hearts. Ventricular fractional shortening was examined as an indicator of cardiac function by transthoracic echocardiography. The presence of diabetic cardiomyopathy in db/db mice was evident by the reduction of fractional shortening. There was a further impairment of cardiac contractile function 7 days after the DOX administration in db/db diabetic mice. According to our microarray analysis, we identified a panel of regulatory genes associated with cardiac remodeling, inflammatory response, oxidative stress, and metabolism in the DOX-induced cardiac injury in diabetic heart. The microarray results of selected genes were confirmed by real time PCR. Notably, S100A8 and S100A9 were found to have a unique specific expression pattern that was coincident with the DOX-induced cardiomyopathy in diabetic hearts. Correspondingly, NF-κB expression in diabetic hearts was increased together with the elevation of S100A8/9 and activation of p38 MAPK signaling after DOX administration, which induced cardiac inflammation as demonstrated by the elevation of cardiac IL-6 level. These findings provide novel pre-clinical information for revealing the S100A8/A9-associated molecular signaling pathways that mediate the doxorubicin-induced cardiotoxicity in diabetic hearts.

## Introduction

Cancer and diabetes mellitus are two leading causes of death in the world according to the figures reported by World Health Organization. Epidemiological studies have evidently demonstrated that there is a strong link between certain cancers and type 2 diabetes mellitus (Giovannucci et al., 2010), although the detailed mechanisms explaining the link are still unclear. Indeed, type 2 diabetes has been shown to increase the risk and mortality of breast, liver, colorectal, and pancreatic cancers (Cannata et al., 2010). Thus, it is not uncommon to see the diagnosis of both cancer and diabetes in the same individual. As the number of people suffering from type 2 diabetes is predicted to be rapidly increasing in the coming decades, it is expected that the number of diabetic cancer patients will also be considerably increasing.

Cancer patients with long standing diabetic cardiomyopathy may present a difficult situation of cancer treatment using chemotherapy. This is attributed to the fact that most of the chemotherapeutic agents cause severe side effects on major vital organs, such as the cardiotoxicity caused by doxorubicin (DOX). DOX is extensively used for treating various cancers, such as breast, stomach, lung, and bladder cancers (Rahman et al., 2007). However, DOX induces life-threatening cardiomyopathy and irreversible cardiac damage (Swain et al., 2003). Chronic conditions, such as diabetes and previous heart disease showed an increased risk of DOX-induced cardiomyopathy (Octavia et al., 2012). Cardiac metabolism is critical in maintaining the myocardial function. In the adult normal heart, 50–70% ATP was obtained from β-oxidation of fatty acids (Lopaschuk et al., 2010), and the remainder being primarily derived from carbohydrate (glucose and lactate) oxidation (Jaswal et al., 2011). However, diabetic hearts showed the increased rates of fatty acid β-oxidation and decreased utilization of glucose (Barsotti et al., 2009; Lopaschuk et al., 2010). Furthermore, defective β-oxidation of fatty acids followed by energy depletion was thought to account for the toxicity of adriamycin in cardiomyocytes (Sayed-Ahmed et al., 2000). In addition, an *in vivo* study found that the accumulation of DOX in the hearts of streptozotocin (STZ)-induced diabetic rats was higher when compared with non-diabetic hearts (Al-Shabanah et al., 2000). It is reasonable to expect that a cancer patient with diabetic cardiomyopathy would be more susceptible to the DOX-induced cardiac damage due to the pre-existing cardiac dysfunction in the diabetic heart.

Currently, the molecular mechanisms underlying the DOX-induced cardiotoxicity in diabetic hearts are largely unknown. It is not clear whether the molecular signaling events that mediate the cardiac toxic effects of DOX on the hearts of diabetic individuals are different from that of non-diabetic individuals. Earlier efforts have proposed a correlation between reduced tissue accumulation of homocysteine and improved cardiac systolic function of diabetic animals treated with peroxisome proliferator-activated receptor gamma (PPAR-γ) agonists (Rocic et al., 2006). Considering the role of homocysteine to promote the assembly of NADPH oxidase associated with endothelial injury (Becker et al., 2005), it is conceivable that DOX might provoke hyperactivation of pro-inflammatory markers in the diabetic myocardium with undefined mechanisms. These pieces of information are essential for developing effective preventive measures and therapeutic strategies to address this complicated clinical situation that occurs in diabetic cancer patients. Thus, this study aimed to investigate the DOX-induced cardiotoxicity in type 2 diabetic hearts. The experiments were designed to distinguish the signaling mechanisms responsible for the DOX-induced cardiotoxic effects on the hearts between diabetic and non-diabetic mice.

## Methods

### Animals

Male 14- to 18-week-old C57BL/KsJ-db/db mice (leptin receptor-deficient transgenic mice which is a well-established animal model of type 2 diabetes mellitus) obtained from the Laboratory Animal Services Centre of The Chinese University of Hong Kong were used in this study. Age-matched non-diabetic db/+ mice with similar genetic background [C57BL/KsJ(+/+)] to db/db mice were used as the non-diabetic control. Mice were housed in a temperature—and humidity-controlled environment and were exposed to a 12:12-h light:dark cycle in the Centralized Animal Facilities of The Hong Kong Polytechnic University. Mice were allowed to have access to standard animal diet and water *ad libitum*. Animal ethics approval (ASESC No. 08/16) was obtained from the Animal Ethics Sub-committee of The Hong Kong Polytechnic University.

### Experimental protocol

Experiment 1: To understand the gene expression profile of the diabetic hearts in response to acute DOX treatment, diabetic (db/db) mice in DOX group were exposed to DOX (Pharmacia and Upjohn SpA, Milan, Italy) at a single dose of 15 mg/kg via an intraperitoneal (i.p.) injection. Diabetic mice in control groups were i.p. injected with the corresponding amount of saline instead of DOX based on the body weight. Mice were euthanized by overdose of ketamine and xylazine 5 days after the administration of DOX. Hearts were immediately removed and washed with cold phosphate buffered saline (PBS). Left ventricle was quickly dissected and frozen in liquid nitrogen and stored at −80°C for later microarray analysis.

Experiment 2: To further reveal the changes of gene expression and the underlying mechanisms in diabetic hearts in response to DOX treatment, mice were treated likewise as in Experiment 1 but sacrificed 5 or 7 days after the administration of DOX. In brief, non-diabetic (db/+) and diabetic (db/db) mice were randomly assigned to the following groups: *db/*+CON, *db/*+DOX-5d, *db/*+DOX-7d, *db/db*CON, *db/db*DOX-5d, and *db/db*DOX-7d (*n* = 6 in each group). Mice in control groups were i.p. injected with the corresponding amount of saline instead of DOX based on the body weight. Mice were euthanized by overdose of ketamine and xylazine 5 or 7 days after the administration of DOX. Left ventricle was quickly dissected and frozen in liquid nitrogen and stored at −80°C for the later molecular analysis.

### RNA extraction and microarray analysis

Forty micrograms of frozen ventricular tissues were minced and homogenized in ice-cold TriReagent (Molecular Research Center, USA). The tissue homogenate was then processed to separation of the aqueous and organic phases. The RNA in the aqueous phase was precipitated by addition of isopropanol followed by washing with 75% ethanol. The extracted RNA pellets were then re-suspended in DEPC-treated water. RNA concentrations were quantified in triplicate using the optical density (OD) at 260 nm. In this study, the technique of microarray analysis was adopted to examine the changes of whole transcriptional profile in response to the administration of DOX in diabetic heart. RNA samples extracted from the ventricular tissues of mice in *db/db*CON and *db/db*DOX groups 5 days after the DOX administration were used in the microarray analysis. RNA samples (3 μg) from the hearts of two mice in each group were pooled to generate four biological replicates in *db/db*CON and *db/db*DOX groups. The RNA quantity and quality were assessed before microarray analysis. RNA quantity was detected by NanoDrop 1000 Spectrophotometer. The purity of RNA was assured by examining the OD260/280 ratio. The RNA integrity was assessed by Agilent 2100 bioanalyzer by following the manufacturer's instruction. In the present study, our RNA samples used for array hybridization showed satisfactory integrity with intact bands corresponding to 18 and 28 S ribosomal RNA and the RNA Integrity Number (RIN) was >7.

Microarray analysis was performed using the Agilent Service Platform with Agilent two-color mouse 4 × 44 k microarray slides. Samples from the DOX-treated diabetic mice (*db/db*DOX) were labeled by Cy5 dye (red channel) whereas samples from diabetic control mice (*db/db*CON) were labeled by Cy3 dye (green channel). Five hundred nanograms of Cy3-labeled and Cy5-labeled cRNA were mixed and incubated with the Agilent microarray slide (G2519F) for 17 h at 65°C in the dark. Slide was washed and scanned using an Agilent DNA microarray scanner. Raw data were obtained using Agilent's Feature Extraction 10.7 Software. Further analysis of the raw data was performed by ArrayTrack comprehensive R—and Bioconductor-based web service for microarray data analysis. Several normalizations were performed for the pre-processing of the raw data and these included background correction by subtraction method, removal of dye bias by lowess normalization and multiple testing correction by BH adjusted *P*-values for the Benjamini and Hochberg step-up FDR controlling procedure (Benjamini and Hochberg, 1995). Gene expression values were calculated by log base 2 ratio of red channel intensity (mean), and green channel intensity (mean). Functional pathways of highly regulated genes were analyzed by MetaCore® (Version 6) from GeneGo Inc.

### Real time quantitative PCR analysis

The transcript levels of target genes from microarray analysis in cardiac muscles were validated by quantitative RT-PCR analysis. Reverse transcription was performed with the PrimeScript™ RT reagent Kit with gDNA Eraser (Takara, Japan) according to the manufacturer's recommendation. One micro litter of the diluted cDNA templates was added to reaction mixtures containing 12.5 μl FastStart Universal SYBR green master (ROX) (Roche Diagnostics, USA), forward and reverse primers and RNase/DNase-free water. The sequences of primers used in real time PCR were shown in Table 1. These mixtures were carried out in the ABI 7500 thermal cycler system followed by a two-step cycling protocol as described previously (Pei et al., 2015). Relative expression ratios were calculated as ratio of the target gene normalized to expression of the internal control β-tubulin gene.

**Table 1:** **Sequence of primer used in real time PCR analysis**.

**Gene**	**Gene full name**	**GenBank accession No**.	**Forward primer**	**Reverse primer**
S100a8	S100 calcium binding protein A8 (calgranulin A)	NM_013650	5′TGCCCTCTACAAGAATGACT3′	5′AAGCTCTGCTACTCCTTGTG3′
S100a9	S100 calcium binding protein A9 (calgranulin B)	NM_009114	5′CGACACCTTCCATCAATACT3′	5′TCAGCATCATACACTCCTCA3′
Il7	Interleukin-7	NM_008371	5′CGCAAGTTGAGGCAA TTTCT3′	5′ TTCCTTGCTTGTGCAGTTCA 3′
Il2r	Interleukin-2 receptor	NM_008368	5′CTGGAGCCTGTCCCTCTACGTCTTCC 3′	5′GACCTGGGAGACCTTCCA GCTTATG3′
Mmp9	Matrix metallopeptidase 9	NM_013599	5′TCCAGTACCAAGACAAAGCC 3′	5′TGA AGCAAA GAAGGAGCCC3′
Mmp8	connective tissue growth factor	NM_008611	5′GGTAACTAACTCTGCAGCCCTCTT3′	5′CGA ACCAGGGACGGA ATATG3′
Wnt5a	Wingless-type MMTV integration site family, member 5A	NM_009524	5′TCCGGACTACTGTGTGC3′	5′AGCAGCACCAGTGAA AC3′
Col1a2	Collagen, type I, alpha 2	NM_007743	5′TGAAGTGGGTCTTCCAGGTCTTTC3′	5′CACCCT TGTTACCGGATTCTCCTT3′
Gsta2	Glutathione S-transferase, alpha 2	NM_008182	5′CCCCTTTCCCTCTGCTGAAG3′	5′TGCAGCCAC ACT AAA ACTTGA AAA3′
Car3	carbonic anhydrase 3	NM_007606	5′GCTCTGCTAAGACCATCC3′	5′ATTGGCGAAGTCGGTAGG3′
Glp2r	Glucagon-like peptide 2 receptor	NM_175681	5′TCATCTCCCTCTTCTTGGCTCTTAC3′	5′TCTGACAGATATGACATCCATCCA C3′
Fabp6	Fatty acid binding protein	NM_008375	5′ACGTGATTGAAAGGGGACGTAACTT3′	5′CATTCTTTGCCA ATGGTGAACTTGT3′
Gys2	Glycogen synthase 2	NM_145572	5′CCAGCTTGACAAGTTCGACA3′	5′ATCAGGCTTCCTCTTCAGCA3′
Igfbp5	Insulin-like growth factor binding protein 5	NM_010518	5′GTTACCCCGCCTCTCTTCC3′	5′TGTCTGAACGTA ACACTATAGAGAGC3′
Ptpn1	Protein tyrosine phosphatase, non-receptor type 1	NM_011201	5′CGGCTATTTACCAGGACATTC3′	5′TGCGGTTGAGCATGACCAC3′
E2f2	E2F transcription factor 2	NM_177733	5′ACGGCGCAACCTACAAAGAG3′	5′GTCTGCGTGTAAAGCGAAGT3′
Sgsm1	Small G protein signaling modulator 1	NM_172718	5′ATGGAGGTGTCCAGCCTGAGAT3′	5′TGGTTTGTGCGTAGCAGGCATG3′
Ube2c	Ubiquitin-conjugating enzyme E2C	NM_026785	5′GGTGACAAAGGAATCTCCGCCT3′	5′GGGAGAGTTTGTACCTCA GGTC3′
β-tubulin	Tubulin, beta	NM_011655	5′CCGGACAGTGTGGCAACCAGATCGG3′	5′TGGCCAAAAGGACCTGAGCGAACGG3′

### Echocardiographic assessment

Cardiac function was assessed before as well as 5 or 7 days after the DOX administration by transthoracic echocardiography by following the procedure described previously (Siu et al., 2007). Animals were anesthetized with ketamine HCl and the echocardiographic images were obtained during the heart rate stabilized at ~500 beats per minute. Ultrasound scanning was performed by Esaote MyLab 70 X-Vision Ultrasound System (Esoate, Italy). Using two-dimensional B-mode and M-mode ultrasonography, left ventricle (LV) end-diastolic, end-systolic dimensions (LVEDD and LVESD), anterior and posterior wall thickness and heart rate were measured according to the leading-edge method of the American Society of Echocardiography (Lang et al., 2006). LV systolic function represented as fractional shortening (FS) and ejection fraction (EF) were calculated by the equation of FS (%) = [(LVEDD − LVESD) / LVEDD] × 100 and EF (%) = Y + [(100 − Y) × 0.15], where Y = [(LVEDD2 − LVESD2) / LVEDD2] × 100 (Dittoe et al., 2007).

### Protein fraction preparation

Protein fractions were extracted from cardiac muscle homogenates as described previously (Siu et al., 2004, 2005). Forty milligrams of frozen hearts were minced and homogenized with a Polytron in ice-cold lysis buffer (10 mM NaCl, 1.5 mM MgCl2, 20 mM HEPES, pH 7.4, 20% glycerol, 0.1% Triton X-100, and 1 mM dithiothreitol). The homogenates were then centrifuged at 875 × g for 5 min at 4°C. The supernatant was collected and subject to further centrifugation three times at 3500 × g for 5 min at 4°C. After the last centrifugation, supernatant was collected as the cytoplasmic protein fraction. This protein fraction with the addition of protease inhibitor (P8340, Sigma-Aldrich) was used in the Western blot analysis and cardiac IL-6 measurement by ELISA. The protein concentrations were detected in duplicate by Bradford assay (Coomassie Protein Assay, Pierce).

### Western blot analysis

Denatured proteins were separated by 10% SDS-PAGE gel and transferred to polyvinylidene difluoride (PVDF) membranes (Immobilon P, Millipore) by using the Bio-Rad Mini Protein II system. Membranes were subsequently blocked by 5% non-fatty milk in PBS with 0.1% Tween-20 (PBST) for 1 h and probed with antibodies against S100A8 (1:500 dilution, Santa Cruz Biotechnology), S100A9 (1:500 dilution, Santa Cruz Biotechnology), phospho-p38 (1:1000 dilution, Cell Signaling Technology), p38 (1:1000 dilution, Cell Signaling Technology), NF-κB (1:500 dilution, Santa Cruz Biotechnology), phospho-ERK1/2 (1:1000 dilution, Cell Signaling Technology), ERK1/2 (1:1000 dilution, Cell Signaling Technology), phospho-Akt (1:1000 dilution, Cell Signaling Technology), and Akt (1:1000 dilution, Cell Signaling Technology) overnight at 4°C. Membranes were then incubated with appropriate secondary antibodies [anti-mouse IgG or anti-rabbit IgG horseradish peroxidase (HRP)-conjugated antibodies (1:5000 dilution, Cell Signaling Technology)] after washing for 1 h. Immunolabeled bands were visualized by the ECL chemiluminescence reaction kit (Perkin Elmer), and captured by Kodak 4000R Pro camera system. Measurement of β-tubulin (1:2000 dilution, Sigma) was included as the internal control reference. The arbitrary units of the blot signal were determined by Image J software and presented as net intensity x band area.

### Measurement of cardiac IL-6 by ELISA

Tissue lysates from ventricular samples were used for the measurement of IL-6 concentration using an ELISA kit (Abcam) according to the manufacturer's instruction. In brief, 10-fold diluted ventricular lysates were added to the reaction wells and incubated overnight at 4°C with gentle shaking. After four times of washing, samples were incubated with biotinylated IL-6 detection antibody for 1 h at room temperature followed by sequent reaction with HRP-streptavidin and one-step substrate reagent. With the addition of stop solution, the intensity of sample reaction was measured at 450 nm. Sample concentration of IL-6 was calculated according to the standard curve generated from the provided standard solutions.

### Statistical analysis

Statistical analysis was performed by using the Statistics Package for Social Science (SPSS) version 11.0. All data were expressed as mean ± standard error of mean (SEM). Two-way ANOVA was used to examine the interaction and main effect(s) of the two experimental factors (i.e., diabetes and DOX). The details of two-way ANOVA (*P*-value, *F*) were provided in the figure (on the top of the figures). When significant differences were observed, statistical comparisons among groups were performed with one-way ANOVA with Tukey's HSD *post-hoc* test. The results of one-way ANOVA were shown in the figure (between the columns). *P* < 0.05 was accepted to be statistically significant.

## Results

### Whole transcriptional profile following DOX exposure in diabetic heart

Gene expression profiles in left ventricular muscle of diabetic mice with and without DOX administration were examined by microarray analysis. In our analysis, only those genes with the transcriptional level changes of 2-fold or higher were identified as significantly regulated. According to our criteria, a total of 709 genes were significantly affected by DOX administration in db/db diabetic heart. Of these, 408 genes were up-regulated and 301 genes were down-regulated by DOX in db/db diabetic heart. Selected gene expressions with significant alteration and the qRT-PCR validation results were shown in Table 2. Furthermore, the significantly regulated genes were selected for further pathway analysis by MetaCore® (Version 6). The top 10 scored pathways (the pathway map with the lowest *P*-value) were identified and presented in Table 3.

**Table 2:** **Significantly regulated genes in response to DOX in diabetic heart (diabetes saline control vs. diabetes 5 days after DOX)**.

**Gene name**	**Microarray (*****n*** = **8)**			**qRT-PCR (*****n*** = **8)**		
	***P*-value**	**Fold change**	**Regulation**	***P*-value**	**Fold change**	**Confirmed**
**INFLAMMATORY/IMMUNE RESPONSE**						
S100 calcium binding protein A8 (calgranulin A)	0.017	5.89	up	0.039	2.82	yes
S100 calcium binding protein A9 (calgranulin B)	0.028	4.08	up	0.09	6.67	yes
Tumor necrosis factor receptor superfamily, member 13c	0.002	2.17	up	0.007	1.62	yes
Interleukin 7	0.004	3.02	up	0.38	1.17	no
Interleukin 2 receptor, gamma chain	0.015	0.45	down	0.68	0.87	no
**CARDIAC REMODELING AND MATRIX**						
Matrix metallopeptidase 9	0.04	2.62	up	0.003	1.86	yes
Matrix metallopeptidase 8	0.016	3.21	up	0.011	4.51	yes
Collagen, type I, alpha 2	0.002	0.43	down	0.31	0.86	no
Wingless-related MMTV integration site 5A	0.002	0.48	down	0.01	0.58	yes
**OXIDATIVE STRESS RELATED**						
Glutathione S-transferase, alpha 2 (Yc2)	0.0004	3.27	up	0.038	1.69	yes
Carbonic anhydrase 3	0.047	0.4	down	0.012	0.72	yes
**METABOLISM**						
Glucagon-like peptide 2 receptor	0.03	3.48	up	0.036	1.72	yes
Fatty acid binding protein 6	0.03	3.31	up	0.018	4.46	yes
Glycogen synthase 2	0.04	2.55	up	0.60	0.84	no
**Akt AND ERK SIGNALING**						
Protein tyrosine phosphatise, non-receptor type 1	0.03	2.02	up	0.031	1.53	yes
**SIGNAL TRANSDUCTION/TRANSCRIPTION PROTEIN**						
E2F transcription factor 2	0.041	0.45	down	0.004	0.25	yes
Ubiquitin-conjugating enzyme E2C	0.008	0.48	down	0.26	0.89	no
Small G protein signaling modulator 1	0.0005	0.42	down	0.119	0.76	no

**Table 3:** **Top 10 pathway maps by MetaCore® analysis in diabetic heart 5 days following DOX**.

**Number**	**Enriched pathway maps**	***P*-value**	**Genes in our data**
1	Cell adhesion_ECM remodeling	1.693E-04	4
2	Transcription_Role of AP-1 in regulation of cellular metabolism	1.102E-03	3
3	Immune response_IL-13 signaling via JAK-STAT	1.691E-03	3
4	Transcription_Androgen Receptor nuclear signaling	1.085E-03	3
5	Immune response_MIF-mediated glucocorticoid regulation	6.201E-03	2
6	Cell adhesion_Endothelial cell contacts by non-junctional mechanisms	7.358E-03	2
7	Proteolysis_Role of Parkin in the Ubiquitin-Proteasomal Pathway	7.358E-03	2
8	Immune response_Antigen presentation by MHC class I	9.940E-03	2
9	Development_Hedgehog and PTH signaling pathways in bone and cartilage development	1.612E-02	2
10	Cytoskeleton remodeling_Cytoskeleton remodeling	1.762E-02	3

### Animal survival analysis

**Figure 1:**
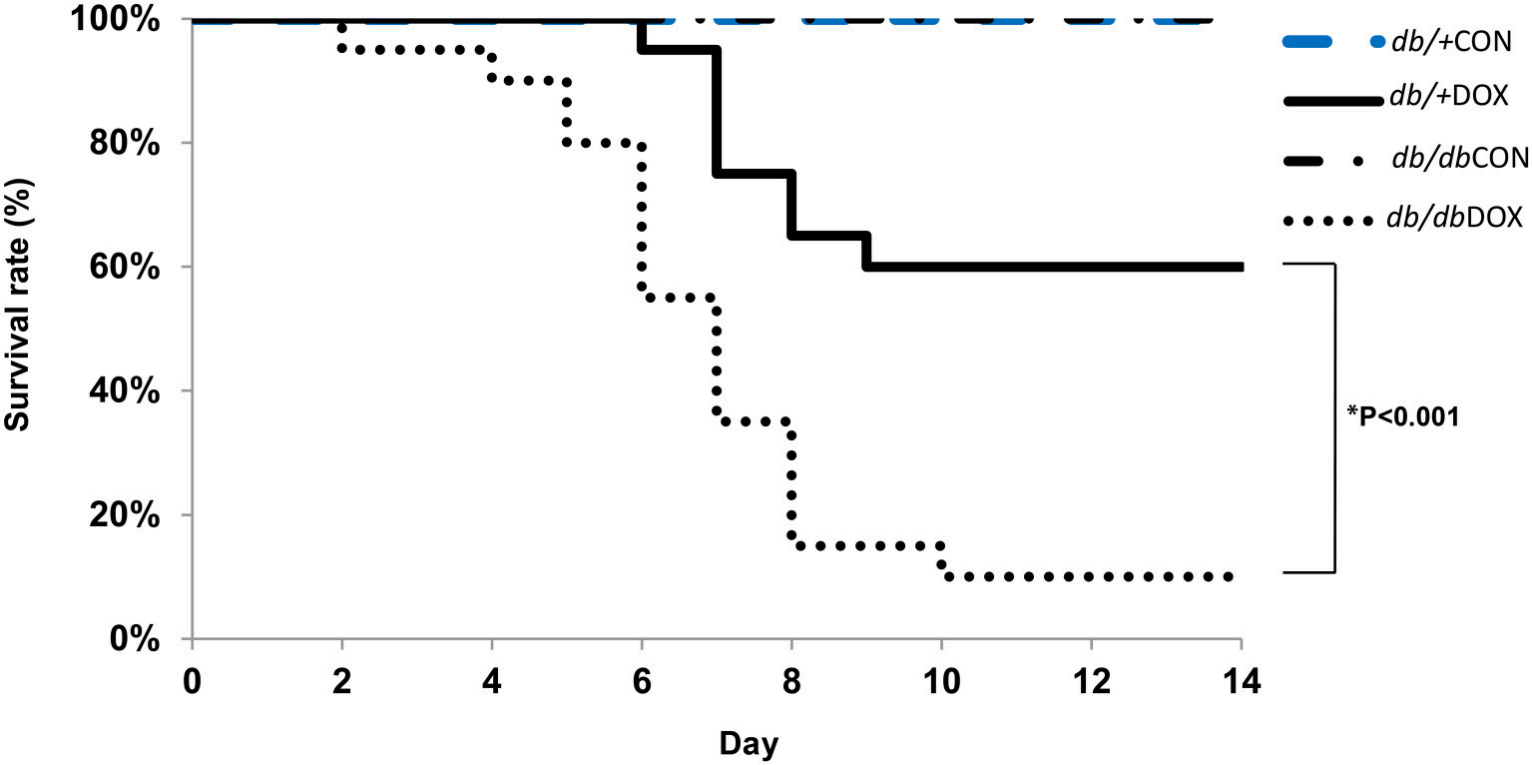
**Animal survival analysis**. Decreased survival rate in *db/*+DOX (non-diabetic mice treated with DOX, *n* = 20 per group), and *db/db*DOX (diabetic mice treated with DOX, *n* = 20 per group). ^*^P < 0.001 compared to non-diabetic mice treated with DOX on day 7 and 14.

The animal survival rates of *db/*+CON (non-diabetic control, *n* = 20), *db/db*CON (diabetic control, *n* = 20), *db/*+DOX (non-diabetic with DOX administration, *n* = 20) and *db/db*DOX (diabetic with DOX administration, *n* = 20) were analyzed by Kaplan-Meier approach (Figure 1). As expected, DOX reduced the survival of both db/+ non-diabetic mice and, db/db type 2 diabetic mice (db/+DOX vs. db/+CON, 60 vs. 100%, *P* < 0.01; db/dbDOX vs. db/dbCON, 10 vs. 100%, *P* < 0.001; Figure 1). However, it is noteworthy that the survival rate of diabetic mice was much lower in comparison with its non-diabetic littermates 7 days after the injection of DOX (35 vs. 75%, *P* < 0.001); this observation was found to be more prominent 14 days after administration (10 vs. 60%, *P* < 0.001).

### Cardiac functional assessment

**Figure 2:**
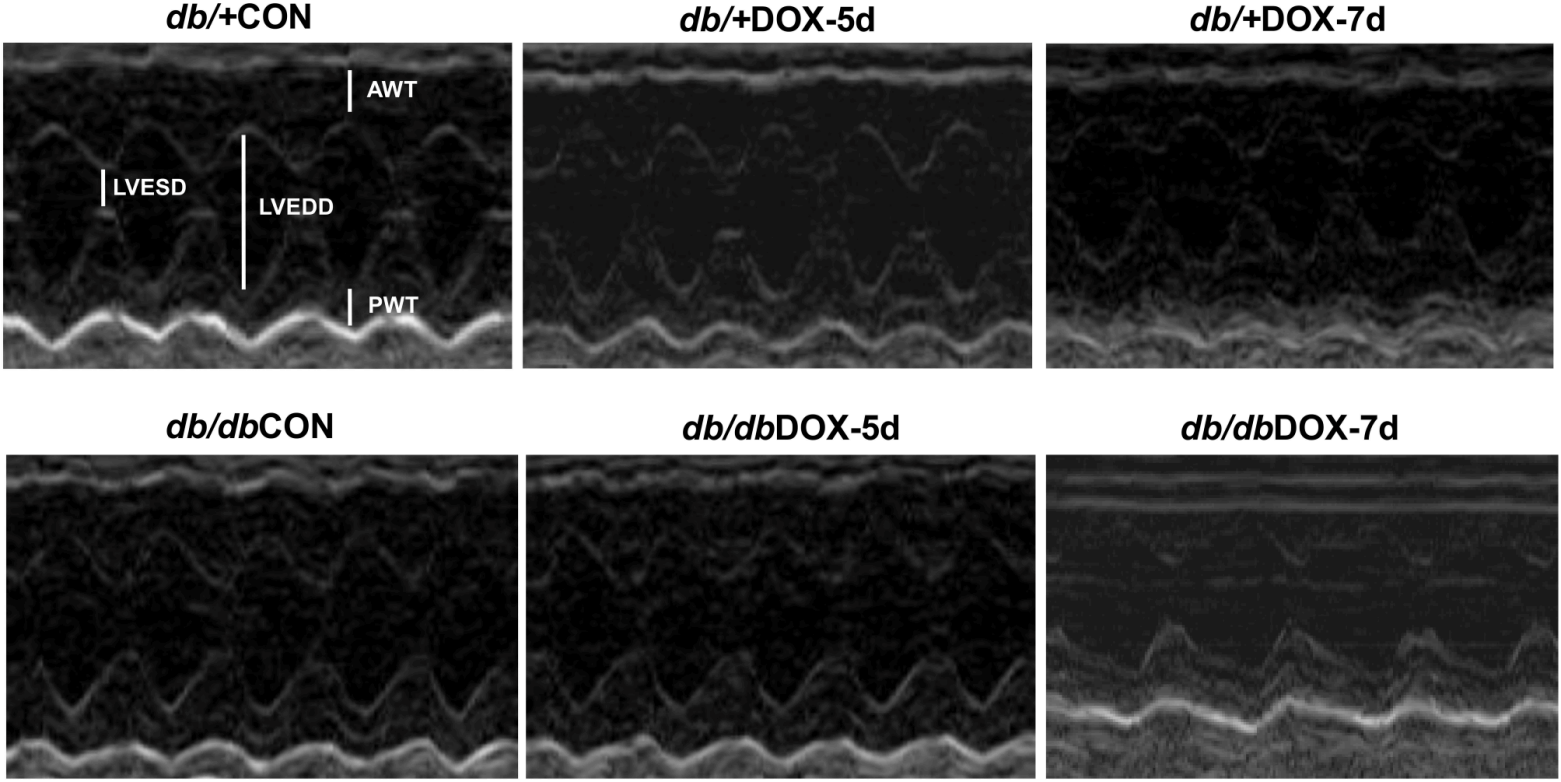
**M-mode echocardiographic image**. Echocardiography of doxorubicin (DOX)-treated non-diabetic and diabetic mice was performed before and after the 5- and 7-day experimental period to assess the cardiac function. *db/*+CON; non-diabetic control group, *db/*+DOX-5d, non-diabetic mice on day 5 with treatment of doxorubicin; *db/*+DOX-7d, non-diabetic mice on day 7 with treatment of doxorubicin; *db/db*CON; diabetic control group, *db/db*DOX-5d, diabetic mice on day 5 with treatment of doxorubicin; *db/db*DOX-7d, diabetic mice on day 7 with treatment of doxorubicin.

Cardiac function was measured non-invasively in non-diabetic and diabetic mice. The representative echocardiograhic M-mode images were shown in Figure 2. DOX induced cardiac dysfunction in db/+ non-diabetic mice 5 days after the administration of DOX as illustrated by a significant reduction of cardiac fractional shortening from 80.9 to 62.4% (*P* < 0.05, Table 4). Cardiac dysfunction resulted from type 2 diabetes was evident by observing a significant decrease in fractional shortening from 81.5% in db/+ non-diabetic mice to 65.7% in diabetic db/db mice at baseline (*P* < 0.05). However, a further decrease in fractional shortening was not observed in db/db diabetic mice 5 days after the administration of DOX. Nonetheless, DOX was found to significantly induce a further impairment of cardiac contractile function in db/db diabetic mice when compared to db/+ non-diabetic mice 7 days after the DOX administration as indicated by a decrease in fractional shortening from 67.3 to 40.3% in the db/db diabetic mice treated with DOX (*P* < 0.05, Table 4). Ejection fraction showed a similar changing pattern with fractional shortening as illustrated by a significant 7.4% decrease in diabetic mice at baseline and a further reduction in diabetic mice 7 days following DOX administration (Table 4).

**Table 4:** **Echocardiographic parameters**.

	***db/+*CON**		***db/+*DOX-5d**		***db/+*DOX-7d**		***db/db*CON**		***db/db*DOX-5d**		***db/db*DOX-7d**	
	**Pre**	**Post**	**Pre**	**Post**	**Pre**	**Post**	**Pre**	**Post**	**Pre**	**Post**	**Pre**	**Post**
HR (bpm)	532 ± 15	503 ± 37	530 ± 24	463 ± 23^*^	563 ± 6	497 ± 29^*^	494 ± 28	503 ± 8	501 ± 20	476 ± 20	485 ± 21	411 ± 32^*^
AWT (cm)	0.10 ± 0.01	0.10 ± 0.01	0.12 ± 0.01	0.10 ± 0.01^*^	0.11 ± 0.01	0.09 ± 0.01^*^	0.10 ± 0.01	0.13 ± 0.02	0.11 ± 0.01	0.11 ± 0.01	0.11 ± 0.01	0.09 ± 0.01^*^
PWT (cm)	0.11 ± 0.01	0.10 ± 0.01	0.10 ± 0.01	0.08 ± 0.01^*^	0.11 ± 0.01	0.09 ± 0.01^*^	0.10 ± 0.01	0.12 ± 0.02	0.12 ± 0.01	0.12 ± 0.01	0.12 ± 0.01	0.10 ± 0.01^*^
LVEDD (cm)	0.35 ± 0.01	0.35 ± 0.01	0.33 ± 0.01	0.34 ± 0.01	0.32 ± 0.01	0.36 ± 0.01	0.35 ± 0.02	0.33 ± 0.01	0.32 ± 0.01	0.33 ± 0.01	0.33 ± 0.01	0.26 ± 0.01^*^
LVESD (cm)	0.07 ± 0.004	0.07 ± 0.01	0.06 ± 0.01	0.13 ± 0.01^*^	0.06 ± 0.002	0.12 ± 0.01^*^	0.12 ± 0.01^**^	0.10 ± 0.006	0.10 ± 0.01	0.11 ± 0.004	0.11 ± 0.01	0.16 ± 0.01^*^
FS (%)	81.5 ± 0.99	80.9 ± 2.11	80.9 ± 1.75	62.4 ± 1.96^*^	82.5 ± 0.75	67.4 ± 1.11^*^	65.7 ± 2.08^**^	70.2 ± 0.92	69.3 ± 2.05	67.3 ± 1.08	67.7 ± 1.66	40.3 ± 4.99^***^
EF (%)	97.0 ± 0.32	96.7 ± 0.65	96.7 ± 0.61	87.8 ± 1.30^*^	97.4 ± 0.22	90.1 ± 0.62^*^	89.8 ± 1.21^**^	91.2 ± 0.46	91.7 ± 1.12	90.8 ± 0.61	91.0 ± 0.91	69.5 ± 5.07^***^

Transthoracic echocardiography was conducted before (Pre) and after (Post) the 5 and 7-day experimental period to examine the ventricular parameters. db/+CON; non-diabetic control group; db/+DOX-5d, non-diabetic mice on day 5 with treatment of doxorubicin; db/+DOX-7d, non-diabetic mice on day 7 with treatment of doxorubicin; db/dbCON, diabetic control group; db/dbDOX-5d, diabetic mice on day 5 with treatment of doxorubicin; db/dbDOX-7d, diabetic mice on day 7 with treatment of doxorubicin. HR, heart rate; AWT, anterior wall thickness; PWT, posterior wall thickness; LVEDD, left ventricular end-diastolic dimension; LVESD, left ventricular end-systolic dimension; FS, fractional shortening; EF, ejection fraction.

* P < 0.05 compared to the corresponding Pre (indicating the effects of DOX treatment on the heart);

** P < 0.05 compared to db/+CON group at Pre (indicating the effects of diabetes on the heart);

*** *P < 0.05 compared to the db/dbDOX-5d Post (indicating the time effects of doxorubicin treatment on diabetic heart)*.

### Body weight loss in diabetic and non-diabetic mice after DOX treatment

Adult db/db diabetic mice are remarkably obese compared to their non-diabetic control. Following the DOX treatment, there were significant body weight loss in both db/db diabetic and db/+ non-diabetic mice (Table 5). After DOX injection, the mean body weight in db/+ mice decreased from 27.4 to 23.9 g on day 5 (by 12%, *P* < 0.01), and to 22.8 g on day 7 (by 16%, *P* < 0.001). Similar trend of body weight loss was observed in db/db diabetic mice treated with DOX (from 47.2 to 43.1 g on day 5 and to 39.6 g on day 7, by 8.7%, *P* < 0.01 and 16%, *P* < 0.001, respectively). However, weight loss was worse in db/db diabetic mice from day 5 to day 7 (by 8.1%) after DOX treatment than db/+ non-diabetic mice (by 4.6%).

**Table 5:** **Body weight at baseline and following DOX treatment**.

**Time (days)**	***db*/+ non-diabetic mice (grams)**		***db/db* diabetic mice (grams)**	
	**CON**	**DOX**	**CON**	**DOX**
Baseline	27.1 ± 1.18	27.4 ± 0.39	45.3 ± 1.74^*^	47.2 ± 1.49^*^
Day 5	26.5 ± 1.02	23.9 ± 0.48^**^	43.3 ± 1.81	43.1 ± 1.80^**^
Day 7	25.4 ± 0.78	22.8 ± 1.39^***^	42.1 ± 1.36	39.6 ± 2.04^***^

Body weights were measured at the baseline and the 5 and 7-day experimental period in db/+ non-diabetic and db/db diabetic mice.

* P < 0.001 compared to db/+ non-diabetic mice at the baseline;

** P < 0.01 compared to the corresponding baseline (indicating the weight loss following DOX treatment on day-5);

*** *P < 0.001 compared to the corresponding baseline (indicating the weight loss following DOX treatment on day-7)*.

### Alteration of specific gene expressions after DOX in diabetic and non-diabetic hearts

**Figure 3:**
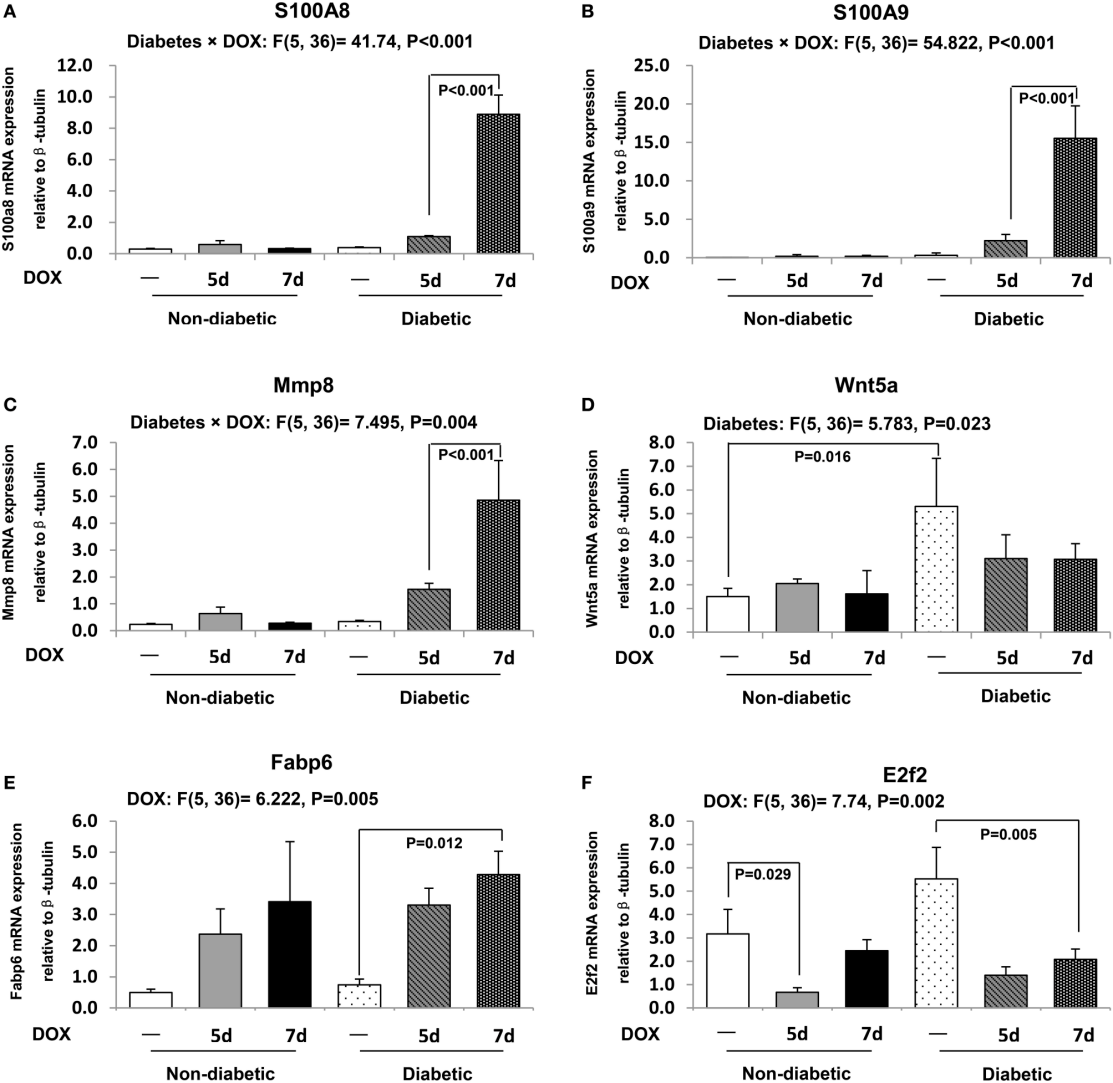
**Transcript expressions of selected genes from microarray results**. The transcript expressions of S100A8 **(A)**, S100A9 **(B)**, Mmp8 **(C)**, Wnt5a **(D)**, Fabp6 **(E)**, and E2f2 **(F)** were examined by real time RT-PCR. Data are presented as expression ratio normalized to β-tubulin gene. Data are expressed as mean ± SEM (*n* = 6 per group). Two-way ANOVA indicated a significant interaction effect between the two experimental factors (i.e., diabetes and DOX treatment) in S100A8 (*P* < 0.001, *F* = 41.74), S100A9 (*P* < 0.001, *F* = 54.822), and Mmp8 (*P* = 0.004, *F* = 7.495; **A–C**). A significant main effect of the factor diabetes was found in Wnt5a (*P* = 0.023, *F* = 5.783; **D**). A significant main effect of the factor DOX treatment was found in Fabp6 (*P* = 0.005, *F* = 6.222), and E2f2 (*P* = 0.002, *F* = 7.74; **E,F**). Individual means were compared among groups by one-way ANOVA with Turkey's HSD *post-hoc* test.

Significant changes of 6 genes including S100A8, S100A9, Mmp8, Wnt5a, Fabp6, and E2f2 as demonstrated by microarray analysis were further confirmed by real time PCR analysis. In addition, the expression levels of these genes were further examined in the hearts of non-diabetic and diabetic mice 5 and 7 days after the DOX administration. The db/+ non-diabetic heart showed a low expression level of S100A8 and S100A9 and no changes were observed 5 or 7 days after DOX treatment (Figures 3A,B). Gene expression of S100A8 was found to be significantly elevated by DOX in diabetic heart 5 days (by 2.8 fold), and 7 days (by 8.2 fold) after DOX administration relative to the diabetic control (Figure 3A). These data suggested that S100A8 might not be involved in DOX-induced cardiotoxicity in non-diabetic mice but was associated with the DOX cardiotoxicity in diabetic heart. Similarly, the transcript level of S100A9 in the heart was increased by DOX in db/db diabetic mice 5 days after DOX administration relative to the diabetic control (by 6.7 fold, *P* < 0.01) and was further elevated by DOX 7 days following DOX administration (by 6.9 fold, *P* < 0.001) when compared to the hearts harvested 5 days after DOX administration (Figure 3B). Transcript expression level of collagenase-2 (Mmp8) was slightly increased in db/+ non-diabetic heart 5 days after DOX administration, but this change did not reach to statistical significance (Figure 3C). Nonetheless, DOX induced an increase in the expression level of Mmp8 5 days (by 4.5 fold, *P* < 0.05) and induced a further increase 7 days following DOX administration (by 3.2 fold, *P* < 0.001) in db/db diabetic mice heart (Figure 3C). Although there was an increased expression of Wnt5a in db/db diabetic heart (by 3.5 fold, *P* = 0.016) and a reduced E2f2 expression in db/+ non-diabetic mice heart 5 days after the DOX administration (by 79%, *P* = 0.029; Figures 3D,E), the gene expression of Wnt5a, Fabp6, and E2f2 were not found to be specifically regulated 7 days following DOX administration in db/db diabetic heart as they showed similar changes as compared to the hearts collected 5 days after DOX treatment (Figures 3D–F).

### Elevation of S100A8/A9 accompanied the activation of p38 MAPK signaling and NF-κB in DOX-treated diabetic hearts

**Figure 4:**
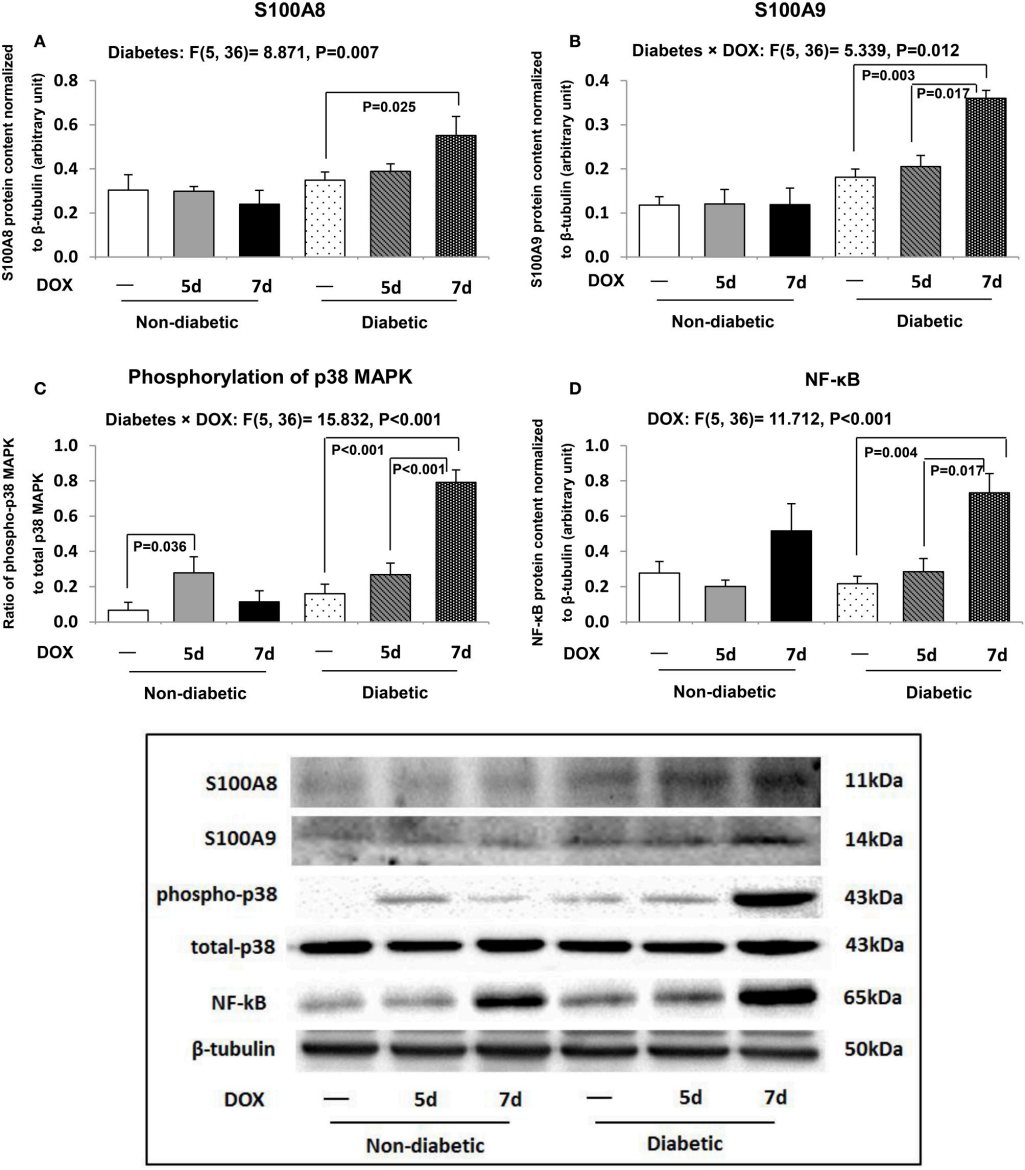
**Cardiac inflammatory pathway**. The protein abundances of S100A8 **(A)**, S100A9 **(B)**, phospho-p38, total p38 **(C)**, and NF-κB **(D)** in the hearts were examined by Western blot. Densitometric quantification was performed and data are presented as net intensity x resulting band area and expressed in arbitrary units. Results are normalized to corresponding β-tubulin signal. Data are expressed as mean ± SEM. According to our two-way ANOVA results, a significant interaction effect between the two experimental factors (i.e., diabetes and DOX treatment) was found in S100A9 (*P* = 0.012, *F* = 5.339; **B**) and phosphorylation of p38 MAPK (*P* < 0.001, *F* = 15.832; **C**). A significant main effect of the factor diabetes was found in S100A8 (*P* = 0.007, *F* = 8.871; **A**). A significant main effect of the factor DOX treatment was found in NF-κB (*P* < 0.001, *F* = 11.712; **D**). Individual means were compared among groups by one-way ANOVA with Turkey's HSD *post-hoc* test.

Consistent with our mRNA findings, the protein abundances of S100A8 and S100A9 in diabetic heart were significantly up-regulated by DOX administration 7 days after DOX administration when compared to db/db diabetic control (Figures 4A,B). S100A8 and S100A9 have been suggested to promote NF-κB activation through p38 MAPK signaling pathway, and therefore, the phosphorylation of p38 and NF-κB were further examined. The p38 MAPK signaling was activated 5 days after DOX treatment in db/+ non-diabetic hearts (by 4.2 fold, *P* = 0.036) when compared to non-diabetic control hearts (Figure 4C). Although this activation of p38 MAPK signaling was not found in db/db diabetic hearts 5 days following DOX administration, phosphorylation of p38 MAPK was activated 7 days after DOX administration in diabetic hearts (*P* < 0.001, Figure 4C). Consistently, the protein abundance of NF-κB was increased (by 2.6 fold, *P* = 0.017) in the hearts of db/db diabetic mice 7 days after DOX treatment when compared to diabetic hearts collected 5 days after DOX administration (Figure 4D).

### Cardiac pro-survival Akt and ERK1/2 signaling and inflammatory marker

**Figure 5:**
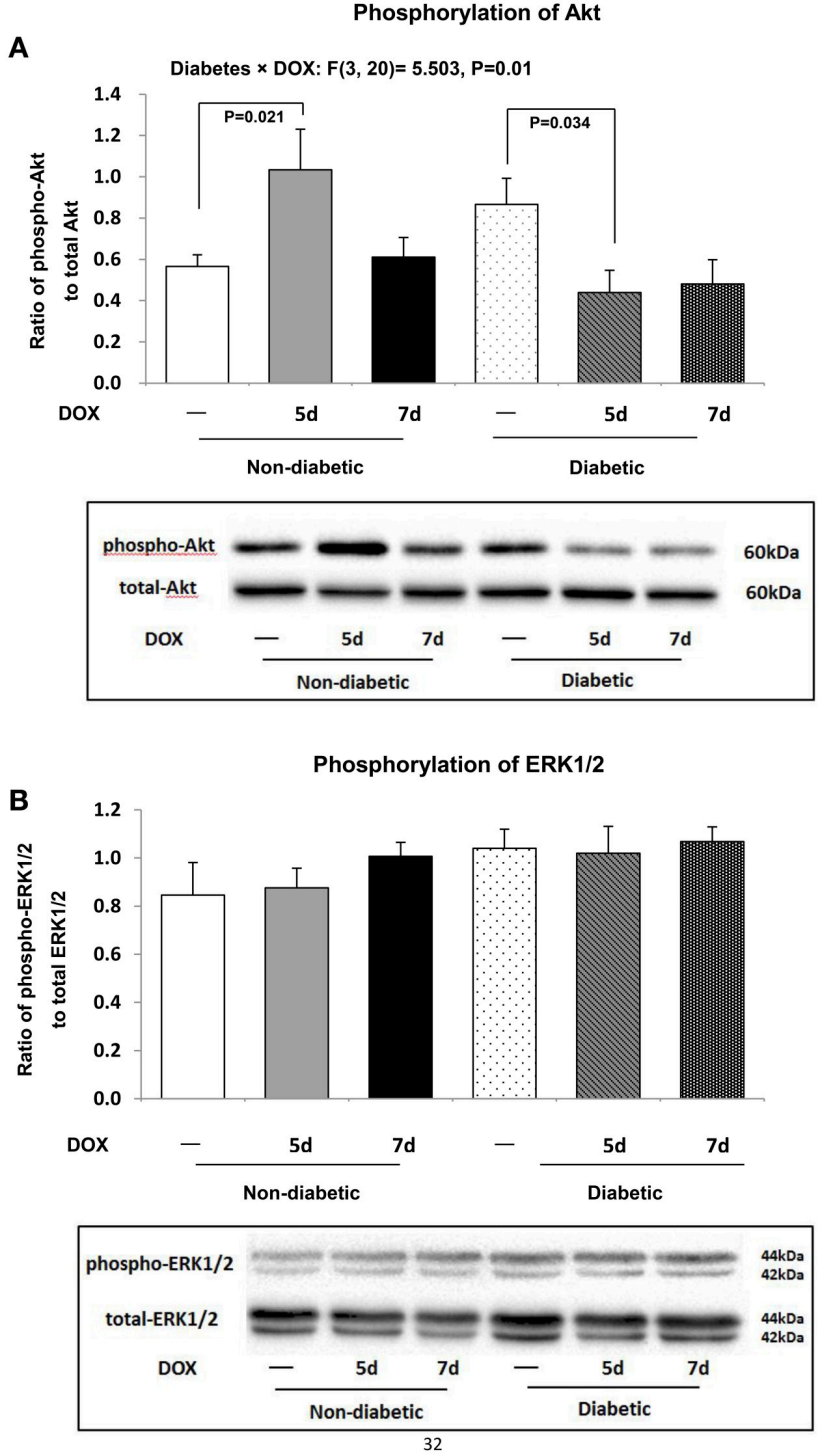
**Myocardial pro-survival cellular signaling**. Activation of Akt and ERK1/2 signaling was determined by examining their phosphorylation statuses. Protein abundances of phospho-Akt, total Akt, phospho-ERK1/2, total ERK1/2 were measured by Western blot analysis. Phospho-Akt-to-total Akt **(A)**, and phospho-ERK1/2-to-total ERK1/2 **(B)** were shown. Data are expressed as mean ± SEM. According to our two-way ANOVA results, a significant interaction effect between the two experimental factors (i.e., diabetes and DOX treatment) was found in the ratio of phospho-Akt-to-total Akt (*P* = 0.01, *F* = 5.503; **A**). Individual means were compared among groups by one-way ANOVA with Turkey's HSD *post-hoc* test.

Pro-survival Akt signaling was activated 5 days after DOX administration in db/+ non-diabetic heart as indicated by an increased ratio of phospho-Akt-to-total Akt (by 1.8 fold, *P* = 0.021) relative to db/+ non-diabetic control mice (Figure 5A). This increase was not observed 7 days after DOX administration in the db/db diabetic mice (Figure 5A). However, db/db diabetic mice 5 days after DOX administration showed a suppression of cardiac Akt signaling (by 49.2%, *P* = 0.034) when compared to diabetic control mice (Figure 5A). DOX did not induce further suppression of the Akt phosphorylation in diabetic heart 7 days after DOX administration (Figure 5A). No significant changes were seen in ERK1/2 signaling in the hearts of mice among all groups (Figure 5B).

**Figure 6:**
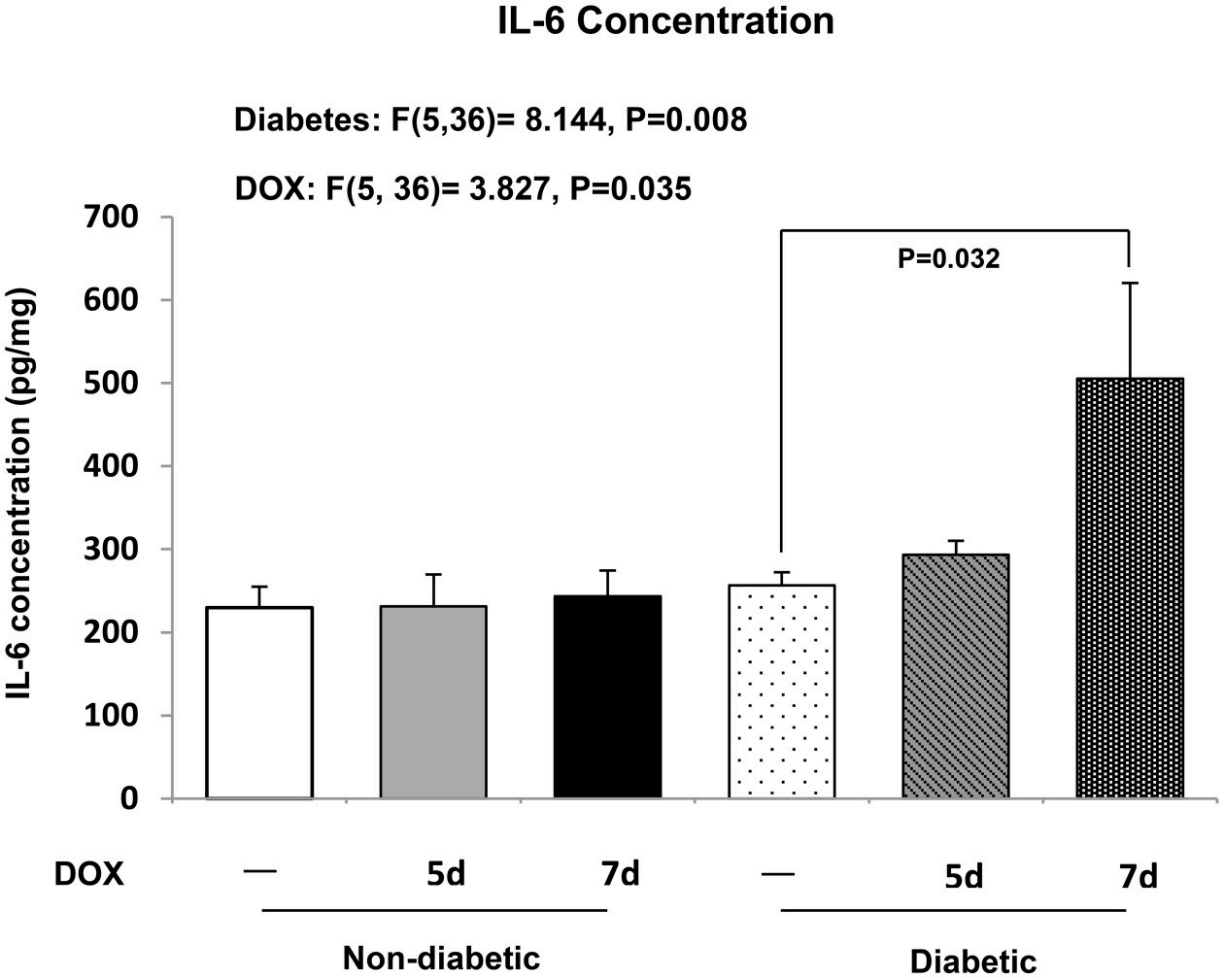
**Cardiac IL-6**. Concentration of IL-6 in the hearts was examined. Data are expressed as mean ± SEM. According to our two-way ANOVA results, the significant main effects of the factor diabetes and DOX were found in IL-6 concentration (Diabetes effect: *P* = 0.008, *F* = 8.144; DOX effect: *P* = 0.035, *F* = 3.827). Individual means were compared among groups by one-way ANOVA with Turkey's HSD *post-hoc* test.

Inflammatory process has been suggested to be involved in the development of S100A8- and S100A9-mediated cardiac dysfunction, and thus the concentration of cardiac IL-6 was examined. IL-6 level in the hearts of db/+ non-diabetic mice was not found to be significantly changed among all groups (Figure 6). However, in the hearts of db/db diabetic mice, there was a significant elevation of cardiac IL-6 level 7 days after DOX treatment (*P* = 0.032) relative to diabetic control mice (Figure 6).

## Discussion

Doxorubicin (DOX) is an effective chemotherapeutic agent for treating many types of cancers including leukemia, cancers of bladder, breast, stomach, and lung. Given that the clinical use of DOX is often accompanied by the development of cardiac failure, its therapeutic value in patients with concomitant presentation of type 2 diabetes is thought to be severely compromised due to the possible aggravation of pre-existing cardiac complications. The acute cardiotoxicity of DOX is considered to be mediated by apoptosis, fibrosis, oxidative stress, and metabolic remodeling (Childs et al., 2002; Suliman et al., 2007; Takemura and Fujiwara, 2007; Yang et al., 2015). Our previous data also showed that DOX induced cardiac dysfunction and apoptosis in non-diabetic mice (Pei et al., 2014). Nonetheless, it is not clear how the diabetic heart is being affected by DOX and these pieces of information are necessary for the exploration of effective strategies to protect the hearts of type 2 diabetic cancer patients undergoing DOX therapy.

In the present study, we hypothesized that the diabetic hearts would be more susceptible to the DOX-induced toxicity compared with non-diabetic hearts. Not only we showed that the basal ventricular fractional shortening was lower in db/db diabetic mice compared with its db/+ non-diabetic littermates, an observation consistent with previous reports (Belke et al., 2004), we have also demonstrated for the first time that this spontaneous reduction in db/db mice was further exacerbated with a dramatic drop of survival rate 7 days after DOX administration. This observation led to the speculation that the molecular mechanisms by which DOX induces cardiotoxicity under diabetic condition were distinct from its counterparts with functional glycemic control. Here, our microarray analyses have unraveled 12 differentially regulated genes including S100A8, S100A9, and Mmp8. in type 2 diabetic hearts challenged with DOX. These data were further validated with quantitative real-time RT-PCR showing consistent findings. Furthermore, our MetaCore® analysis revealed that the significantly regulated genes were related to the regulation of cell adhesion (extracellular matrix remodeling), transcription of AP-1 in regulation of cellular metabolism, immune response of IL-13 signaling via the JAK/STAT pathway, and transcriptional event of androgen receptor nuclear signaling.

S100 calcium binding protein A8 (S100A8), and A9 (S100A9) are members of the S100 family implicated in inflammatory response and immune diseases (Belke et al., 2004; Ehrchen et al., 2009). Recent human studies have suggested that S100A8/A9 levels were elevated in type 2 diabetic patients (Peterson, 2002; Ortega et al., 2012) and indeed, the S100A8/S100A9 complex could be an useful biomarker for the prediction of 1-year mortality in elderly patients with severe heart failure (Ma et al., 2012). Moreover, an *in vivo* study showed that the activation of S100A8/A9 was crucial for the development of post-ischemic heart failure via the innate immune receptors including receptor of advanced glycation end products (RAGE), and toll-like receptor 4 (TLR4) (Harja et al., 2008; Volz et al., 2012). Notably, the expression of S100A8 and S100A9 was increased abruptly at both transcript and protein levels in diabetic hearts 7 days after DOX challenge. Previous reports have shown that the cardiac overexpression of S100A8/A9 was associated with decreased calcium flux and ejection fraction (Boyd et al., 2008) whereas knockdown of S100A9 ameliorated the lipopolysaccharide-induced cardiac dysfunction (Boyd et al., 2008). Furthermore, administration with a S100A9-neutralizing antibody was found to prevent the activation of NF-κB, inflammatory cell infiltration, cytokine production, subsequent perivascular and interstitial fibrosis, and hypertrophy in the heart induced by angiotensin II infusion (Wu et al., 2014). Taken together, our novel data suggest that pharmacological blockade of S100A8/A9 may represent a promising cardioprotective strategy in cancer patients with history of diabetes, thus restoring the therapeutic value of doxorubicin in these patients.

Next, we investigated the mechanisms that are related to S100A8 and S100A9 for the detrimental effects of DOX in diabetic hearts. The effects of S100A8/A9 on the downstream signaling were shown to be varied in different models. For instance, p38 MAPK and ERK1/2 was shown to be activated and suppressed, respectively, following the binding of S100A8/A9 to RAGE in gastric cancer cells (Kwon et al., 2013) whilst in colon cancer cells, S100A8/A9 induced the activation of ERK1/2 and JNK but not p38 MAPK (Ichikawa et al., 2011). While S100A8/A9-RAGE binding increased the activities of p38 MAPK, JNK, ERK1/2, and NF-κB in isolated ventricles from a murine model of post-ischemic heart failure (Volz et al., 2012), S100A8/A9 failed to activate Akt/ERK signaling and subsequent inflammation and fibrosis in necrotic myocardial cells of TLR4-deficient mice (Zhang et al., 2015). This is the first attempt reporting that S100A8/A9 elevated the phosphorylation level of p38 MAPK in the heart of db/db diabetic mice. In agreement with the critical role of ERK1/2, but not p38 MAPK, in the promotion of cardiac hypertrophy (Peterson, 2002; Rose et al., 2010; Asrih et al., 2013), neither did doxorubicin provoke the activation of ERK1/2 nor development of hypertrophy in diabetic hearts. More prominently, the induction of p38 signaling was known to trigger the release of pro-inflammatory cytokines including IL-6, TNFα, and IL-1β through the action of NF-κB (Rose et al., 2010; Schiopu and Cotoi, 2013; Simard et al., 2013; Gao et al., 2015). Provided that NF-κB mediated the pro-inflammatory effects ofS100A8/A9 (Vogl et al., 2012) and the suggestion of S100A8/S100A9 complex and IL-6 as markers of prognosis in senior patients with heart failure (Ma et al., 2012), we observed concordantly that the protein abundances of NF-κB and IL-6 were increased in diabetic hearts in response to DOX exposure.

**Figure 7:**
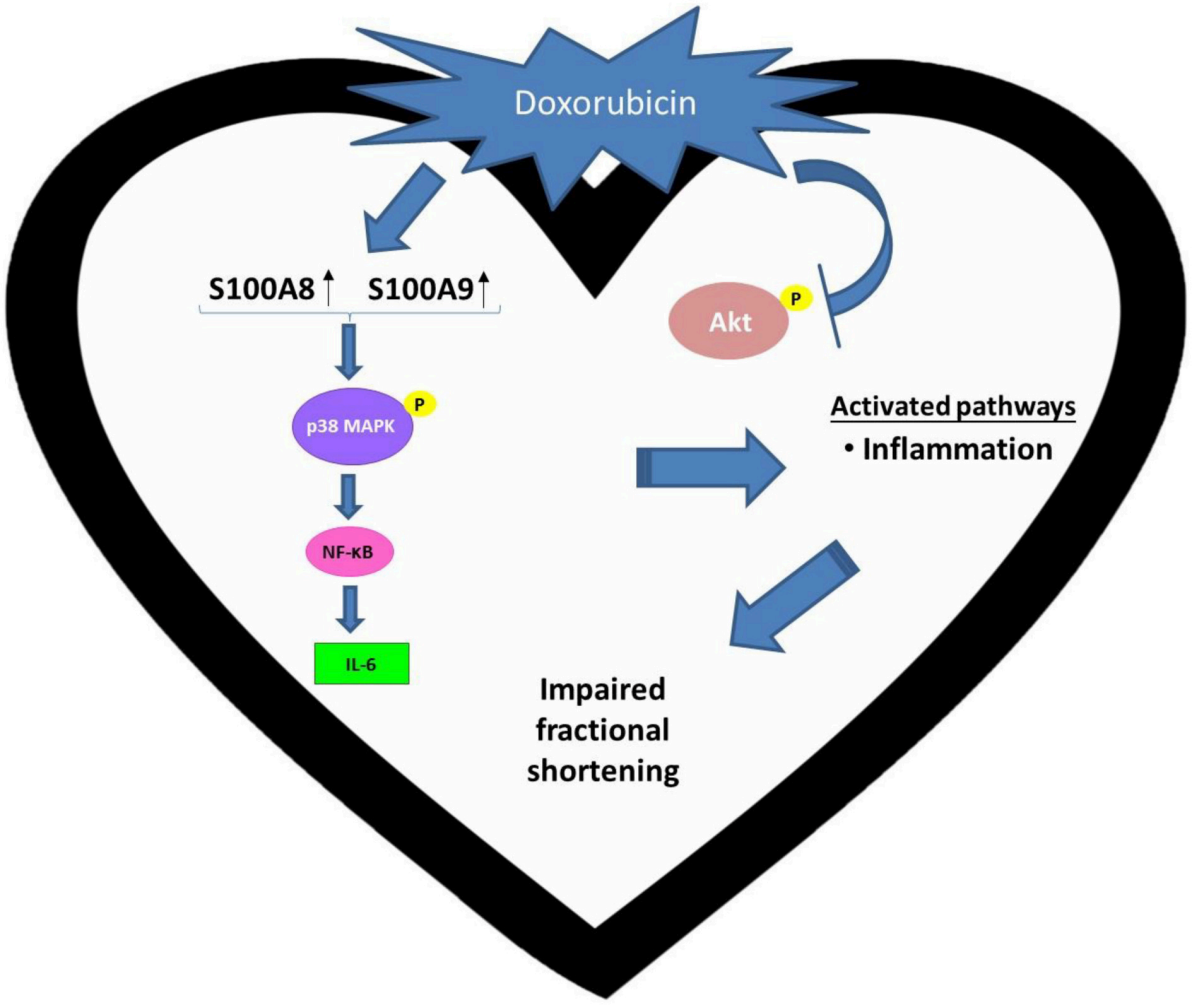
**Distinct mechanism of doxorubicin-induced toxicity in diabetic hearts**. Doxorubicin (DOX) up-regulates S100A8/A9 and increases the expression of IL-6, which is a pro-inflammatory cytokine, in the diabetic heart through the p38 MAPK/NF-κB axis. It is noteworthy that these observations were not made with non-diabetic hearts subject to DOX challenge.

Collectively, the present work is the first to reveal two damage-associated molecular pattern (DAMP) proteins, S100A8 and S100A9, are associated with the doxorubicin-induced cardiotoxicity in type 2 diabetic hearts. Distinct mechanism of doxorubicin-induced toxicity in diabetic hearts was summarized in Figure 7. We propose that the increased expression of cardiac NF-κB and IL-6 and activation of p38 MAPK may underpin S100A8/A9-mediated inflammation as a potential target for the attenuation of myocardial toxicity in type 2 diabetic individuals receiving doxorubicin therapy.

## Author contributions

Conceived and designed the experiments: XP, BY, LC, CW, CL, PS; Performed the experiments: XP, BT, TS; Analyzed the data: XP, FW; Contributed reagents/materials/analysis tools: FW, MY; Contributed to the writing of the manuscript: XP, PS.

### Conflict of interest statement

The authors declare that the research was conducted in the absence of any commercial or financial relationships that could be construed as a potential conflict of interest.
